# Breed distributions for diabetes mellitus and hypothyroidism in Norwegian dogs

**DOI:** 10.1186/s40575-022-00121-w

**Published:** 2022-05-24

**Authors:** N. K. Ringstad, F. Lingaas, S. I. Thoresen

**Affiliations:** 1 Department of Companion Animal Sciences, Faculty of Veterinary Medicine, Norwegian University of Life Sciences, Ås, Norway; 2 Department of Preclinical Sciences and Pathology, Faculty of Veterinary Medicine, Norwegian University of Life Sciences, Ås, Norway

**Keywords:** Canine, Diabetes mellitus, Hypothyroidism, Breed distribution, Autoimmune endocrinopathies

## Abstract

**Background:**

Diabetes mellitus (DM) and hypothyroidism are common canine endocrinopathies. Both canine DM and primary hypothyroidism are assumed to originate from autoimmune destruction of the respective endocrine glands and have been associated with the major histocompatibility complex (MHC) gene region. This study aims to investigate breed distributions for DM and hypothyroidism in the Norwegian canine population by calculating odds ratios (OR) from two different comparator groups.

**Methods:**

Results from canine serum samples submitted from 2001 to 2018 to the Veterinary Clinical Pathology Laboratory (VCPL) at the Faculty of Veterinary Medicine, Norwegian University of Life Sciences for analysis of fructosamine and thyroid hormones in serum were used as cases in a retrospective bivariate analysis of canine breeds. The ORs were calculated as a measure of risk for the included breeds, where all the submitted blood samples to the VCPL and dogs registered in the Norwegian Kennel Club (NKK), the national organization for dog owners, were used as two comparator groups.

**Results:**

Significant differences in disease prevalence between breeds were discovered using both comparator groups. Australian terrier, Swedish lapphund, Samoyed, and Schipperke were at highest risk for DM. German Shepherd, Golden retriever, German pointing dog, and Collie presented as the breeds with lowest risk for DM. For hypothyroidism, Schnauzer, Eurasier, Dunker, and English setter were at highest risk for developing the disease. The breeds at lowest risk of developing hypothyroidism were Rottweiler, Dachshund, German shepherd, and Border collie. The results from the different comparator groups gave different ORs and ranks, but the breeds with highest and lowest odds showed the same susceptibility using both comparators.

**Conclusions:**

These findings support that there are breeds more and less prone to develop DM and hypothyroidism. A strong genetic predisposition involved in the aetiology of these two diseases is therefore likely. Interestingly, there also appeared to be an inverse relationship of odds for the two diseases for some of the breeds since some breeds that had a high OR for DM or hypothyroidism had a lower OR for the other disease. This indicates that there may be different risk alleles/haplotypes for the two diseases. The possible aetiological relationship between canine DM and hypothyroidism should be further investigated.

## Plain English summary

Diabetes mellitus (DM) and hypothyroidism are diseases that are caused by dysregulations in the hormone system and are categorized as endocrine disorders. Diabetes mellitus and hypothyroidism are chronic diseases and may strongly affect the quality of life for the affected dogs if the treatment fails. One of several acknowledged mechanisms behind both diseases are associated with autoimmunity, where the immune system attacks and destructs the respective hormonal gland. Previous studies have investigated other possible factors that might influence the development of the diseases, including sex, breed and weight.

Breed differences were discovered for DM and hypothyroidism in the present study. The most susceptible breeds for DM were the Australian terrier and Swedish lapphund, whilst German Shepherd and Golden Retriever were the least susceptible. Schnauzer and Eurasier where the most susceptible for hypothyroidism, while Rottweiler and Dachshund were the least susceptible. There were indications of an opposite relationship between the two diseases in some breeds as some of the breeds showed high susceptibility for one of the diseases, and low susceptibility for the other disease. Other breeds seemed to be susceptible for both diseases. This information supports a multigenetic complex inheritance and could be of importance in future genetic studies of these diseases.

## Background

The endocrine system is vital for normal body function, with dysfunction potentially leading to severe clinical implications [1]. Endocrinopathies diverge in severity depending on the affected gland, but most of these diseases are chronic and require lasting treatment [2, 3]. Canine endocrinopathies can therefore affect the quality of life for both the dog and the owner. In several canine endocrinopathies the immune system play an important role in the aetiology [4] and are commonly referred to as autoimmune endocrinopathies.

In canines, two of the most commonly occurring endocrinopathies are DM and hypothyroidism. Diabetes mellitus is a disease characterized by persistent hyperglycemia due to impaired response to insulin, or impaired ability to produce insulin. The persistent hyperglycemia leads to increased formation of fructosamine (1-amino-1-deoxy-d-fructose), a glycated protein formed by the non-enzymatic, irreversible Amadori-rearrangement between glucose and the amino group of proteins, a compound that is utilized for diagnosis and monitoring of DM in dogs [2]. Accumulation of glycated proteins (Advanced Glycation End products, AGEs) affect nearly every type of cell and molecule in the body and might cause severe damage to the cardiovascular system, eyes, kidneys, and nerves [5]. In canines, DM can present with a variety of clinical signs, however, the clinical signs most commonly recognized are related to impaired metabolism, such as polyuria, polydipsia, polyphagia, and weight loss [2, 4].

The classification of canine DM has been discussed and changed over the last decades. Previously, the terms insulin-dependent DM (IDDM) and non-insulin-dependent DM (NIDDM) were commonly used [1]. In dogs, IDDM resembles type 1A DM (T1ADM) diagnosed in humans, where the body causes autoimmune destruction of the insulin-producing β-cells in the pancreas [6]. Evidence of a serological autoantibody reaction to pancreatic β-cell proteins has been reported in dogs as well [7–10]. In contrast to T1ADM in humans that mainly occur during childhood, the disease has a later onset in dogs, with a peak prevalence between 7 and 10 years [4, 11]. The aetiologic classification into insulin deficient DM and insulin resistant DM made by ESVE (European Society of Veterinary Endocrinology) is considered the preferred classification system today [12]. Compared to the old IDDM classification, the new classification with type 1B (insulin deficient DM) is more accurate in canines.

Hypothyroidism is caused by insufficient production or antibody inactivation of thyroid hormones [13]. In dogs, this is most often the result of an autoimmune response on the thyroid gland with lymphoid infiltration into the gland, also called lymphocytic thyroiditis categorized as primary hypothyroidism [4]. This will cause an irreversible loss of thyroid tissue, and the dog will need enduring thyroid hormone replacement therapy. Hypothyroidism can also be caused by a pituitary neoplasia resulting in inadequate thyrotropin (TSH)-production and hence, an underactive and histologically atrophic thyroid gland. This is categorized as central or secondary hypothyroidism. Clinical signs of hypothyroidism are non-specific and may be subtle, such as tiredness, alopecia, weight gain, and cold intolerance. These clinical signs reflect the functions of the thyroid hormones as a metabolic actor [14, 15]. In clinical hypothyroidism, the disease is characterized by elevated serum TSH-concentrations and decreased concentrations of free thyroxine (FT4) and total thyroxine (TT4). As in humans, there is probably subclinical hypothyroidism also in dogs, characterized by only elevated TSH-concentrations or thyroglobulin autoantibodies (TgAA) in serum [15–17].

Both DM and hypothyroidism are assumed to be complex (multifactorial) diseases caused by genetic, epigenetic and environmental factors in dogs [18, 19]. Several studies have indicated that some canine breeds have a genetic predisposition for the diseases [20–22], and many possible aetiological risk factors have been investigated [22, 23]. Sex, weight, and age are acknowledged factors that may influence risk for DM [23, 24]. The prevalence of DM is reported to be significantly higher for female dogs in countries where elective spaying is not allowed [6]. Progesterone stimulates local canine mammary growth hormone production which contributes to systemically clinically overt insulin resistance during metoestrus in some dogs [4, 25]. In a study from the UK where spaying is elective, no significant sex-predisposition was discovered [19], and the annual prevalence is estimated to be around 0.3% (1 of 300 dogs) in the UK [11, 19, 26]. In an epidemiological study from Australia, the prevalence of DM in dogs was reported to be 0.36% per year [27]. For several decades the predisposition of some breeds to canine DM has been investigated and reported. Especially Samoyed and Australian terrier have frequently been reported at high risk of development [18, 19, 21, 22, 27–29] and the German Shepherd and Boxer at low risk [11, 18, 22, 26–30]. Other studies have indicated that other breeds are at high risk in some countries, e.g. the Irish Setter and English Setter in Italy [30]. These differences could be due to demographic differences in environment or allele frequencies between breeds in different countries.

For hypothyroidism, no sex-predisposition has been shown, although this has been a topic for discussion in many epidemiologic studies [4, 31, 32]. The influence of risk factors in the development of canine hypothyroidism is sparsely known [17]. Unfortunately, the diagnostic criteria have varied between studies for canine hypothyroidism, making it difficult to conclude on breed distribution. However, several studies have indicated that English Setter, Doberman, Rhodesian Ridgeback, Gordon Setter, and Giant Schnauzer are at higher risk of developing lymphocytic thyroiditis and hence, hypothyroidism [33, 34].

Autoimmune diseases are assumed to originate from defects in certain antigen-presenting genes. Both canine DM and hypothyroidism have been associated with the MHC class II region [10, 15, 20, 21, 34–37], but other candidate genes have also been investigated [14, 38]. Certain haplotypes of the MHC class II region have been connected to protection and susceptibility for DM in several breeds [10, 20, 21]. In canine hypothyroidism, especially the DLA-DQA1*001:01 allele has been associated with risk of the disease in some breeds [15, 34, 37]. The two diseases are both commonly diagnosed in dogs and can occur in the same individual dog [39–41].

The present study aimed to provide more information concerning breed predispositions of canine DM and hypothyroidism based on data from the Norwegian canine population. The objective of the study was to describe relative differences in breed prevalence for canine DM and hypothyroidism to substantiate potential genetic influence in the aetiology of these diseases. The null hypothesis was therefore that there are no differences in breed prevalence for DM and hypothyroidism, and the alternative hypothesis being that there are differences in prevalence for breeds in the two diseases.

## Results

### Overall laboratory results

**Fig. 1:**
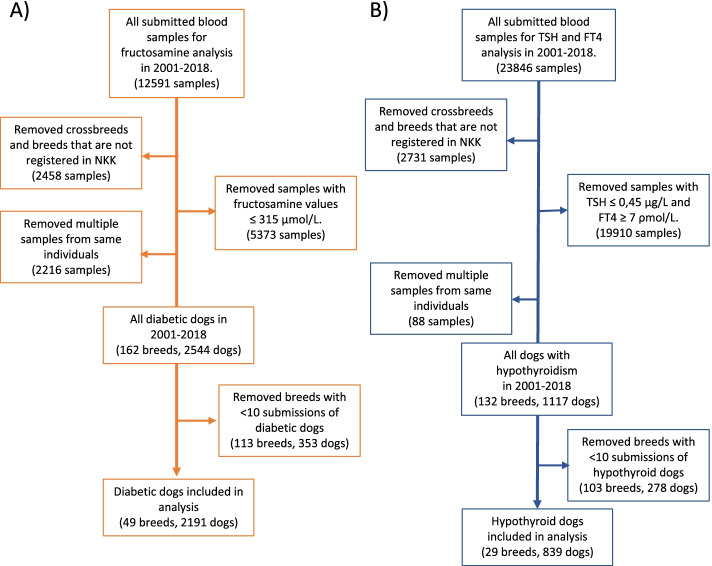
**A** Inclusion of dogs with fructosamine concentrations compatible with diabetes mellitus. **B** Inclusion of dogs with TSH and Free T4 concentrations compatible with hypothyroidism

The database consisted of clinical pathology results from 212,732 canine blood samples submitted for analysis from 2001 to 2018. Fructosamine results were obtained from 12,591 serum samples from unique dogs representing 49 breeds submitted from 2001 to 2018. Fructosamine results from 2191 (17.4%) of these blood samples were classified as compatible with diabetes mellitus according to the given diagnostic criterium of persistently elevated serum glucose concentrations identified by an elevated serum fructosamine concentration (Fig. 1A). The average age of the DM cases in this study was 8.8 years old. The median age of the cases was 9, where the ages varied from under 1 year old to 18 years old. Approximately 62% of the DM cases were females, with some variation between breeds. The average age and percentage of females per breed is listed in Table 1.

**Table 1:** Breed distribution and OR for diabetes mellitus (fructosamine > 315 μmol/L)

Breed	No. of cases	Odds Ratio A: VCPL	Odds Ratio B: NKK	Ranks A - B	No. of dogs in CG: A - B	Mean age of cases in the breed	% Females
Australian Terrier	54	11.4 (8.2–15.6)^**^	11.0 (8.1–14.6)^**^	1st - 1st	218–680	8.6 (7.9–9.3)	57%
Swedish Lapphund	37	8.2 (5.5–11.8)^**^	7.7 (5.3–10.7)^**^	2nd -2nd	192–646	8.9 (8.1–9.7)	52%
Samoyed	63	4.5 (3.4–5.9)^**^	3.2 (2.5–4.2)^**^	3rd -7th	547–2545	9.1 (8.5–9.7)	70%
Schipperke	10	3.6 (1.7–6.9)^**^	3.5 (1.6–6.5)^**^	4th -6th	104–367	9.9 (8.4–11.4)	50%
Keeshond	16	3.2 (1.8–5.4)^**^	5.7 (3.2–9.4)^**^	5th -4th	185–366	9.3 (8.1–10.5)	57%
Finnish Hound	54	2.7 (2.0–3.6)^**^	2.0 (1.5–2.7)^**^	6th -10th	728–3414	7.4 (6.8–8.1)	59%
Hamiltonstövare	16	2.6 (1.5–4.4)^**^	1.6 (0.9–2.7)^*^	7th -12th	222–1240	7.1 (5.9–8.4)	64%
West Highland White Terrier	40	2.6 (1.8–3.6)^**^	6.1 (4.3–8.4)^**^	8th -3rd	570–868	9.5 (8.7–10.3)	47%
Border Collie	193	2.6 (2.2–3.0)^**^	1.6 (1.3–1.8)^**^	9th -14th	2872–16,150	9.2 (8.9–9.6)	88%
Norwegian Buhund	23	2.5 (1.5–3.8)^**^	1.7 (1.1–2.6)^**^	10th -11th	331–1700	10.0 (9.0–11.0)	95%
Basenji	14	2.5 (1.3–4.2)^**^	2.8 (1.5–4.7)^**^	11th - 8th	207–649	10.2 (8.9–11.5)	56%
Bichon Frise	149	2.3 (2.0–2.8)^**^	3.5 (3.0–4.2)^**^	12th -5th	2381–5692	9.5 (9.1–9.9)	60%
Finnish Lapponian dog	26	2.2 (1.4–3.3)^**^	1.4 (0.9–2.0)^NS^	13th -21st	427–2425	9.5 (8.6–10.5)	44%
Jämthund	40	2.2 (1.5–3.0)^**^	1.1 (0.8–1.5)^NS^	14th -23rd	667–4697	8.0 (7.2–8.7)	94%
English Setter	259	2.1 (1.8–2.4)^**^	2.5 (2.2–2.8)^**^	15th -9th	4719–14,216	9.2 (8.9–9.5)	62%
Cairn Terrier	55	1.8 (1.3–2.3)^**^	1.6 (1.2–2.1)^**^	16th -13th	1111–4308	9.5 (8.9–10.2)	43%
Miniature Poodle	37	1.6 (1.1–2.2)^**^	1.4 (1.0–2.0)^*^	17th -18th	835–3305	9.3 (8.5–10.1)	60%
Lagotto Romagnolo	10	1.5 (0.7–2.8)^NS^	0.8 (0.4–1.6)^NS^	18th -28th	239–1475	9.6 (8.1–11.1)	57%
Tibetan Terrier	17	1.4 (0.8–2.3)^NS^	1.5 (0.9–2.4)^NS^	19th -16th	417–1457	8.2 (7.0–9.4)	36%
Japanese Spitz	19	1.4 (0.8–2.3)^NS^	0.9 (0.5–1.4)^NS^	20th -27th	470–2756	8.0 (6.8–9.2)	38%
Newfoundland	10	0.3 (0.2–0.6)^**^	0.6 (0.3–1.1)^NS^	45th -38th	1003–2142	7.3 (5.8–8.8)	50%
Collie	13	0.3 (0.2–0.5)^**^	0.4 (0.2–0.8)^**^	46th -43rd	1392–3671	6.8 (5.5–8.2)	57%
German Pointing Dog	21	0.3 (0.2–0.4)^**^	0.4 (0.2–0.6)^**^	47th -45th	2468–7053	9.8 (8.7–10.9)	80%
Golden Retriever	49	0.3 (0.2–0.4)^**^	0.4 (0.3–0.5)^**^	48th -44th	5730–15,324	7.3 (6.6–8.0)	59%
German Shepherd	18	0.1 (0.05–0.1)^**^	0.1 (0.1–0.2)^**^	49th -49th	6273–19,999	7.1 (5.9–8.3)	69%

The table shows the odds ratio per breed calculated with the two comparator groups. The confidence interval of the OR is set to 95% and is marked within the parenthesis. The number of cases per breed and the rank for each breed within the different comparator groups are listed. *P*-values for the OR calculations are marked with * ≤ 0.05, ** ≤ 0.01, and NS (not significant) > 0.05. Comparator group A consist of the total submitted blood samples for dogs for any reason to VCPL, and comparator group B consist of new NKK registrations. Both comparator groups consist of registrations from 2001 to 2018, and the number of dogs in the comparator groups (CG) are listed. The mean age of the cases and % females in the breeds are listed in the table

During the same period, there were 23,846 submitted canine serum samples for TSH and FT4 analysis. For the hypothyroid cases, there were 29 breeds included when breeds represented with less than 10 individuals were excluded. A total of 839 submitted serum samples were classified as compatible with hypothyroidism from these 29 breeds (Fig. 1B). The average age of the hypothyroid cases was 6.7 years old, and the median age was 7 years. The age varied from 2 years old to 14 years old. The case group for hypothyroidism consisted of 55% females. The average age and percentage of females per breed is listed in Table 2.

**Table 2:** Breed distribution and OR for hypothyroidism (FT4 < 7 ρmol/L, and TSH > 0.45)

Breed	No. of cases	Odds Ratio A: VCPL	Odds Ratio B: NKK	Ranks A - B	No. of dogs in CG: A - B	Mean age of cases in the breed	% Females
Eurasier	45	8.4 (6.0–11.5)^**^	5.7 (4.1–7.7)^**^	1st - 1st	443–2068	5.2 (4.5–5.9)	59%
Schnauzer	12	6.2 (3.1–11.3)^**^	2.0 (1.0–3.5)^*^	2nd -7th	147–1504	5.7 (4.3–7.1)	55%
Dunker	15	2.1 (1.2–3.5)^**^	1.5 (0.8–2.5)^NS^	3rd - 10th	528–2513	4.5 (3.1–5.8)	65%
English Setter	124	2.1 (1.7–2.5)^**^	2.4 (1.9–2.9)^**^	4th - 4th	4719–14,216	7.8 (7.3–8.3)	44%
Portuguese Water Dog	11	2.0 (1.0–3.7)^*^	1.3 (0.7–2.4)^NS^	5th - 11th	395–2040	6.3 (4.9–7.6)	60%
Gordon Setter	96	1.9 (1.5–2.4)^**^	2.1 (1.7–2.6)^**^	6th - 6th	3882–11,956	7.8 (7.3–8.3)	57%
Finnish Lapponian Dog	11	1.9 (0.9–3.4)^*^	1.1 (0.6–2.0)^NS^	7th - 18th	427–2425	5.0 (3.4–6.6)	67%
American Cocker Spaniel	27	1.9 (1.2–2.8)^**^	2.3 (1.5–3.3)^**^	8th - 5th	1058–3003	6.6 (5.7–7.6)	56%
Jämthund	17	1.9 (1.1–3.0)^**^	0.9 (0.5–1.4)^NS^	9th - 21st	667- 4697	6.1 (4.8–7.4)	57%
Bichon Havanais	11	1.8 (0.9–3.3) ^*^	0.6 (0.3–1.0)^NS^	10th - 24th	445–4619	6.7 (5.3–8.1)	56%
English Cocker Spaniel	60	1.8 (1.4–2.4) ^**^	1.9 (1.4–2.4)^**^	11th - 8th	2492–8177	6.9 (6.3–7.6)	60%
Kleiner Münsterländer	11	1.7 (0.8–3.1)^NS^	2.5 (1.3–4.6)^**^	12th - 3rd	468–1081	5.6 (4.2–7.0)	67%
Shetland Sheepdog	42	1.5 (1.1–2.1)^**^	1.3 (0.9–1.8)^NS^	13th - 12th	1995–7950	6.5 (5.7–7.2)	38%
Giant Schnauzer	30	1.5 (1.0–2.2) ^*^	4.2 (2.8–6.1)^**^	14th - 2nd	1436–1818	6.7 (5.8–7.5)	43%
Alaskan Malamute	15	1.5 (0.8–2.5)^NS^	1.2 (0.7–2.0)^NS^	15th - 14th	739–3079	5.4 (4.0–6.7)	38%
Labrador Retriever	28	0.4 (0.3–0.6)^**^	0.6 (0.4–0.9)^**^	25th - 23rd	4663–11,501	6.5 (5.5–7.4)	55%
Border Collie	16	0.4 (0.2–0.6)^**^	0.2 (0.1–0.4)^**^	26th - 28th	2872–16,150	7.3 (6.1–8.4)	56%
German Shepherd	29	0.3 (0.2–0.4)^**^	0.3 (0.2–0.5)^**^	27th - 25th	6273–19,999	5.6 (4.8–6.5)	74%
Dachshund	16	0.3 (0.2–0.5)^**^	0.3 (0.2–0.5)^**^	28th - 27th	3742–13,022	7.4 (6.3–8.6)	36%
Rottweiler	12	0.2 (0.1–0.4)^**^	0.3 (0.2–0.5)^**^	29th - 26th	3726–9643	6.4 (5.0–7.8)	55%

The table shows the odds ratio per breed calculated with the two comparator groups. The confidence interval of the OR is set to 95% and is marked within the parenthesis. The number of cases per breed and the rank for each breed within the different comparator groups are listed. *P*-values for the OR calculations are marked with * ≤ 0.05, ** ≤ 0.01, and NS (not significant) > 0.05. Comparator group A consist of the total submitted blood samples for dogs for any reason to VCPL, and comparator group B consist of new NKK registrations. Both comparator groups consist of registrations from 2001 to 2018, and the number of dogs in the comparator groups are listed. The mean age of the cases and % females in the breeds are listed in the table

Comparator group 1 consisted of 136,761 unique blood samples in total. Comparator group 2 consisted of 454,385 newly registered dogs to the NKK in total.

### Odds ratio for diabetes mellitus

The calculated OR for the 20 breeds at highest rank and the 5 with lowest rank for DM is shown in Table 1. Australian Terrier presents with the highest odds and the Swedish Lapphund, Samoyed, and West Highland White are also within the 10 highest ranked using both comparator groups. The breeds with lowest odds for DM in this dataset were German Shepherd, Golden Retriever, German Pointing Dog, Collie, and Newfoundland. Breeds such as Boxer, Chihuahua, and Bernese Mountain Dog were not included in the list as they did not have ≥10 DM-cases registered during the period.

For the two comparator groups there are differences in the ranks for many of the breeds. The two comparator groups are however in accordance with each other regarding the breeds at highest and lowest rank for the disease.

### Odds ratio for hypothyroidism

For hypothyroidism, Eurasier, Schnauzer, English Setter, Dunker, and Gordon Setter are the breeds with high risk using both comparator groups (Table 2). The Giant Schnauzer also scores high using comparator group B. The breeds with lowest OR for hypothyroidism were Rottweiler, Dachshund, German Shepherd, Border Collie, and Labrador Retriever. As with DM, several popular breeds did not meet the inclusion criteria and are therefore likely at low risk of hypothyroidism. This includes the Tibetan Spaniel, Irish Setter, Staffordshire Bull Terrier, and Bernese Mountain Dog. These breeds, with 3, 7, 7, and 3 cases respectively, comprised 4.7% of all samples submitted to VCPL during the study period.

The two comparator groups showed a greater spread in OR and ranks for the breeds with high odds of hypothyroidism. The results from the two comparators were more in accordance with each other on the breeds at lowest rank.

### Both diseases

**Fig. 2:**
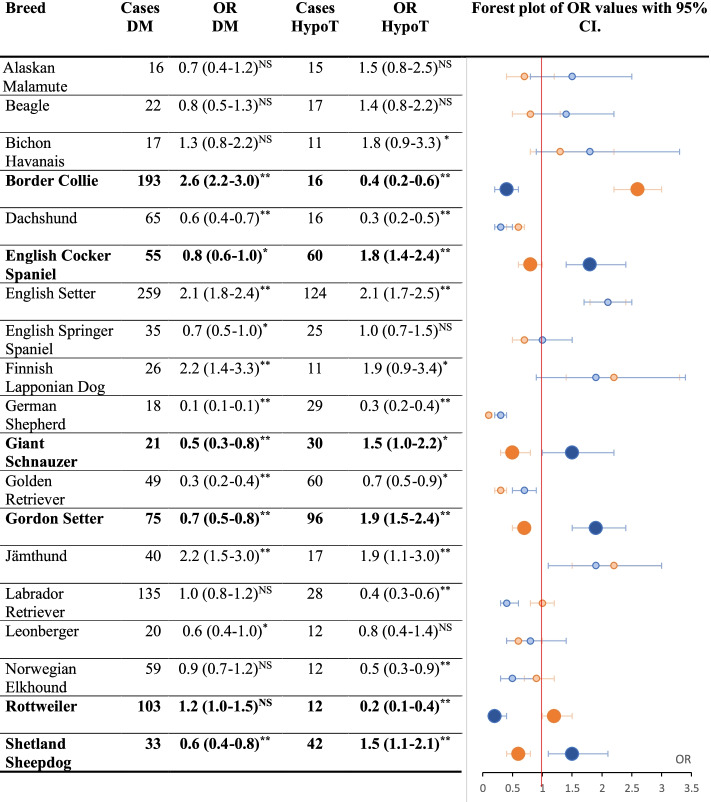
Odds ratio (OR) values for diabetes mellitus and hypothyroidism with a corresponding forest plot. OR values with 95% confidence interval of the breeds that had ≥10 cases for both diseases with a corresponding forest plot. *P*-values for the OR calculations are marked with * ≤ 0.05, ** ≤ 0.01, and NS (not significant) > 0.05. Diabetes mellitus is marked with orange and hypothyroidism is marked with blue in the forest plot. The dots express the OR, and the lines represent the 95% confidence interval. The breeds highlighted with bold text and marked dots in the forest plot show a tendency of an opposite risk for the two diseases. The number of cases for the diseases per breed are listed in the table

Figure 2 presents the OR of the breeds that had ≥ 10 cases for both diseases. The highlighted breeds show a tendency of opposite risk for the two autoimmune diseases. Some breeds show a low OR for both diseases, and some breeds have a high OR for both diseases.

There were 15 (0.7%) dogs with both DM and hypothyroidism in this study: five English Setters (33.3%), two Alaskan Malamute (13.3%), two Bichon Havanais (13.3%), two Giant Schnauzer (13.3%), two Samoyed (13.3%), one Border Collie (6.7%), and one Leonberger (6.7%).

## Discussion

The present study support that there are breed differences in the prevalence of canine DM and hypothyroidism. For some breeds, such as the Australian Terrier, Samoyed, and West Highland White Terrier the results of this study coincide with previous findings of predisposed breeds for DM [11, 18, 19, 21, 22, 26–29]. The breeds with highest odds of developing hypothyroidism in this study are the Eurasier, Dunker and English Setter which also is in accordance with earlier studies [33, 34].

The breeds with the lowest odds for DM were German Shepherd, Golden Retriever and German Pointing Dog. Amongst the popular breeds that were expected to meet the inclusion criteria of ≥10 cases were Boxer, Staffordshire Bull Terrier, Chihuahua, and Bernese Mountain Dog. The fact that these four breeds did not meet the inclusion criteria of ≥10 cases during these 18 years, supports that they are likely at very low risk of developing DM. Especially Boxer, German Shepherd, and Golden Retriever have frequently been reported to be at low risk in many studies [11, 18, 22, 26–30].

Breeds with the lowest odds of hypothyroidism were the Rottweiler, Dachshund and German Shepherd. The Tibetan Spaniel, Irish Setter, Staffordshire Bull Terrier, and Bernese Mountain Dog are also likely at low risk of developing hypothyroidism as these breeds did not meet the inclusion criteria.

It is interesting to observe an inverse odds for the two diseases for some of the breeds. English Cocker Spaniel, Gordon Setter, Giant Schnauzer, and Shetland Sheepdog presented with higher odds for hypothyroidism and lower odds for DM. The inverse tendency, high odds for DM, and low odds for hypothyroidism, was seen in Border Collie and Rottweiler. The English Setter, on the other hand had high odds for both DM and hypothyroidism in this study. This might support a polygenetic predisposition and that the risk of the two diseases is influenced by the presence/absence of risk alleles in several genes/chromosomal regions including the MHC region. The fact that breeds may have different risk in different countries is also to be expected in genetic disorders with a multifactorial aetiology. Allele frequencies of risk alleles may vary from population to population within the same breed as a results of different population sizes. The interesting part of studying breed distributions is not only which breeds that are at risk, but that these breed differences exist, and that the differences in diseases-associated allele frequencies between breeds provide an excellent opportunity to identify the genes with functional effects.

The possible confounding effect of age and sex was not assessed for each breed, and this could be a limitation to the study. In Tables 1 and 2 the average age and percentage of females for the breeds with high and low odds of the disease is presented. The average age and percentage of females were similar in the five breeds at highest and lowest rank in both diseases. We believe that the results from the highest and lowest ranked breeds are therefore not influenced by age or percentage of females, but truly are a result of a genetic or environmental predisposition/protection in these breeds. The overall percentage of females in the DM cases (62%) was lower than expected. The lack of neuter status in the dogs make these results difficult to explain.

In this study the cases were diagnosed through laboratory data, and no clinical data were available to support the status of cases and the controls. The inclusion criteria for DM and hypothyroidism were set to deviations from the VCPL’s reference intervals for the analyses relevant for the diagnoses. The reference interval used for each of the tests are based upon 95% of the results in a healthy population. A low number of the dogs in the case group could therefore in theory not be diseased. However, the dogs with samples for fructosamine, FT4 and TSH were probably requested based on clinical implication for running the respective tests, supporting the laboratory diagnosis.

Fructosamine concentrations in serum between 315 μmol/L and 350 μmol/L indicate poor glycemic control commonly seen in an early stage of DM. Normal variation in the concentration of fructosamine is also shown to be associated with specific loci [42]. The majority of the cases in our study had fructosamine concentrations in serum above 350 μmol/L, which is strongly associated with DM in dogs [43]. The sensitivity and specificity of serum fructosamine for canine diabetes in dogs with clinical signs of the disease is high [44, 45]. Serum fructosamine is considered as a reliable test for canine diabetes mellitus, and a single positive fructosamine result from dogs with clinical signs usually equate to a veterinarian diagnosing the dog with diabetes mellitus.

Hypothyroidism in dogs can be challenging and time-consuming to diagnose as the clinical signs are unspecific, very subtle and develop gradually. Due to this, the clinical implications of analysis of thyroid hormones are more unspecific than for DM. The diagnostic criteria for canine hypothyroidism have partly changed over time and between laboratories, so the comparison between studies may be influenced by analytic methodology. A decrease in both TT4 and FT4 concentrations combined with elevated TSH concentration in the same sample is considered to have a specificity > 90% for diagnosing hypothyroidism [46, 47]. This was used as the basis for the inclusion criteria in this study and should exclude “euthyroid sick” canines.

There are several advantages of using laboratory data, such as the accessibility of large dataset. The blood samples in the study were not sampled for scientific purposes and the study did not cause any extra harm or stress for the dogs included in the analysis.

In this study an inclusion criterion of ≥10 cases was applied. This criterion was added to avoid coincidental findings, especially from small breeds with few cases and very few individuals in the comparator groups. By adding this criterion some of the breeds with few cases were not included in the study, such as the boxer that has frequently been reported with low risk of DM. This is a limitation to the study, but we do believe that the inclusion criterion of ≥10 cases is necessary to avoid uncertain results from small breeds with few cases.

Unfortunately, data on the number of dogs of each breed, are not available. In the present study, we have therefore used two comparator groups as the best approximation to the “real population size”. Compared to an ideal control population, the comparator groups used in this study could be influenced by unknown factors such as geographics. Comparator group A was all samples submitted for any reason per breed in the same period to the VCPL. The numbers for each breed may theoretically be influenced by a potential risk of other specific diseases that is diagnosed by clinical pathology. We are not aware of examples of such bias, and we believe that breed distribution of the total number of samples received in general is an acceptable alternative to adjust for the population at risk for each breed. We believe that the breed classification in this comparator group is reliable for pedigreed dogs. There is however a risk of duplicates in this comparator group if a dog is registered with multiple owners, under different names or registered with many typographical errors in the database. To account for potential bias in the total-sample-received method we also used the number of dogs recorded per breed in the Norwegian Kennel Club (NKK) in the same period to adjust for differences in breed population size. The data from NKK are highly reliable in regards of breed classification. The diseases in question in this study primarily affect middle-aged to older dogs [4, 11]. The data from the kennel club consist of new registrations to the breed from 2001 to 2018. Dogs would usually be affected by the diseases within a few years of age, and there is no reason to believe that breed popularity and trends compared would change very fast within such a short period.

We do believe that the relative breed distribution represents the overall relative breed popularity as the data for both cases and comparators is gathered over a long time period. The scope of this study was to investigate differences in breed predispositions as an indication of accumulation of risk alleles in these breeds. Due to the lack of knowledge about the “breed content” of crossbreeds, they were excluded from the study population.

## Conclusion

These results support that there are breeds more and less susceptible of developing DM and hypothyroidism supporting a genetic predisposition for DM and hypothyroidism. The breeds with inverse risk for the two diseases should be of special interest in such genetic studies, especially as both diseases are associated with the same chromosomal.

## Methods

### Study population

In this study, the database generated at the Veterinary Clinical Pathology Laboratory (VCPL), Faculty of Veterinary Medicine, Norwegian University of Life Sciences, containing results from submitted canine blood samples from January 1. 2001 to December 31. 2018 were used. This database contains clinical pathology results from 212,732 canine blood samples, submitted from all over Norway for diagnostic purposes. Veterinary clinics submitting blood samples to the VCPL were informed that the sample also could be used for scientific research. Information concerning breed, age and sex was available for the cases but not for the comparator groups. Duplicates from individual dogs were excluded. Some of the closely related breeds were combined as such breeds are considered genetically similar. This affected the Chihuahua (long-, and shorthaired), Collie (long-, and shorthaired), Dachshund (long-, short-, and wirehaired), Giant Schnauzer (black and salt/pepper), Miniature Schnauzer (white, salt/pepper and black), Norwegian Elkhound (black and grey), Schnauzer (black and salt/pepper) and Welsh Corgi (Pembroke and Cardigan) in this study. Crossbreeds and breeds not registered as official breeds in the Norwegian Kennel Club (NKK) were not included in the analysis. To exclude coincidental findings, only breeds represented by at least 10 records in the database were included.

### Comparator groups

In this study we used to different comparator groups for both diseases as an estimation to the real Norwegian canine population. The comparator groups were breed specific and we only included the breeds with cases for the respective disease. The individuals in the comparator groups were not assessed/verified as true controls.

Comparator group A consisted of all blood samples submitted to VCPL from 2001 to 2018 after duplicates were removed. In comparator group A information on breed was available. For DM this comparator group consisted of 76,128 unique blood samples from 49 breeds. For hypothyroidism this comparator group consisted of 60,100 blood samples from 29 breeds.

Comparator group B consisted of new breed registrations from 2001 to 2018 in the Norwegian Kennel Club (NKK). The Norwegian Kennel Club is the main organization for dog owners in Norway where most dogs are registered. Only breed information was available from this comparator group. For DM this comparator group consisted of 274,839 registrations from 49 breeds. Comparator group B for hypothyroidism consisted of 206,517 registrations from 29 breeds.

### Blood sample analysis

Samples analysed for serum fructosamine were used for diagnosing DM, and analysis of TSH and FreeT4 in serum were used for diagnosing hypothyroidism based on defined criteria. The criterium for classifying a diabetic dog was a serum fructosamine concentration > 315 μmol/L. Fructosamine was analysed in serum by the Siemens Advia® 1800 Clinical Chemistry System (Siemens Healthcare GmbH, Germany) using the nitrobluetetrazolium-chloride (NBT) analytical method (Horiba Medical). For hypothyroidism, the criteria for classifying primary hypothyroidism in a dog were a serum TSH-concentration > 0.45 μg/L and a free thyroxine concentration (FT4) < 7 ρmol/L in the same sample. TSH and FT4 were analysed in serum by the Siemens Immulite® 2000 Immunoassay System using chemiluminescence methods (Siemens Healthcare GmbH, Germany). The laboratory has used the same analytical methods, analyzers and reference ranges during the whole period.

### Odds ratio calculations

The OR was calculated as the odds of having the respective disease for each breed and hence, ranked to all the other breeds that fulfilled the inclusion criteria. Each OR was calculated with a two-sided 95% confidence interval. Two groups were used as comparators in the OR calculation, see more information about these under *comparator groups.* The OR was calculated with the same cases for the two comparator groups. The breeds were ranked in both groups from highest to lowest OR. Calculations of odds ratio (OR) for the breeds were made using Excel® (Microsoft Corporation) and Stata®SE 16 (StataCorp LLC, USA).

## Data Availability

The datasets analysed during the current study are not publicly available due to privacy of dog owners that could not be fully anonymized. The datasets are available from the corresponding author on reasonable request.
